# Optimization of Functional Group Concentration of N, N-Dimethylacrylamide-based Polymeric Coatings and Probe Immobilization for DNA and Protein Microarray Applications

**DOI:** 10.3390/mi14020302

**Published:** 2023-01-24

**Authors:** Laura Sola, Laura Abdel Mallak, Francesco Damin, Alessandro Mussida, Dario Brambilla, Marcella Chiari

**Affiliations:** Institute of Chemical Sciences and Technologies “G. Natta”, National Research Council of Italy-CNR, Via Mario Bianco, 920131 Milano, Italy

**Keywords:** functional polymers, probe immobilization, surface functionalization, protein microarray, DNA microarray

## Abstract

We report here a deep investigation into the effect of the concentration of a polymeric coating’s functional groups on probe density immobilization with the aim of establishing the optimal formulation to be implemented in specific microarray applications. It is widely known that the ideal performance of a microarray strictly depends on the way probes are tethered to the surface since it influences the way they interact with the complementary target. The *N*, *N*-dimethylacrylamide-based polymeric coating introduced by our research group in 2004 has already proven to offer great flexibility for the customization of surface properties; here, we demonstrate that it also represents the perfect scaffold for the modulation of probe grafting. With this aim in mind, polymers with increasing concentrations of *N*-acryloyloxysuccinimide (NAS) were synthesized and the coating procedure optimized accordingly. These were then tested not only in DNA microarray assays, but also using protein probes (with different MWs) to establish which formulation improves the assay performance in specific applications. The flexibility of this polymeric platform allowed us also to investigate a different immobilization chemistry—specifically, click chemistry reactions, thanks to the insertion of azide groups into the polymer chains—and to evaluate possible differences generated by this modification.

## 1. Introduction

The development of biosensors is attracting more and more attention from researchers since the easy, fast and cost-effective qualitative and quantitative identification of biological species has tremendous impact in several fields, ranging from medicine and pharmaceutics to food, environment and biotechnologies [1,2,3,4]. Generally, biosensors comprise a probe interface which recognizes and binds to a target molecule; this interaction is then transformed into a quantifiable analytical signal by a transducer [2,5].

One of the main features that assures sensitivity, stability, and specificity to biosensors is an appropriate modification of the sensor surface [6]. In fact, the proper functionalization of a sensor area assures correct binding of the analytic probe in terms of space, orientation and activity retention, promoting the recognition by its counterpart and maximizing their interactions [6,7]. Moreover, the appropriate surface modification drastically reduces non-specific interactions of other molecules mediated by hydrophobic and electrostatic interactions. These features directly influence the specificity and sensitivity of the assay, minimizing background signal and false positive signals. The grafting density of probes at the sensing interface is another critical parameter impacting the performance of biosensors since it provides possible binding sites for the analyte, but it also affects the accessibility of the analyte toward the probe surface by the occurrence of steric and electrostatic effects [8,9].

The literature reports countless approaches suited to surface modification purposes. In particular, polymeric coating is one of the most widely exploited techniques, since polymers extend into the third dimension, generating high cross-sectional densities of functional groups homogeneously distributed onto the surface, therefore resulting in an increased binding capacity of probes [10,11]. Moreover, polymeric coating offers several other advantages, such as the facility in tailoring their composition to adapt the coating to specific applications or material, high accessibility of the ligand for the interaction with the partner, lower degree of non-specific adsorption (meaning reduced background signal) and exceptional retention of the ligand 3D structure, all of which consequently improve a probe’s biological activity. With the aim of controlling probe density, Movilli and collaborators have proposed two strategies based on the deposition of modified poly-(l-lysine) polymers with various ratios of oligo(ethylene glycol) (OEG) maleimide (Mal) moieties (PLL-OEG-Mal) or clickable groups (in particular, dibenzocyclo-octyne (DBCO)) able to couple oligonucleotides [12,13]. However, these approaches suffer from the scarcity of stability and homogeneity offered by the interaction of poly-(l-lysine) with the surface, which is merely electrostatic; moreover, the formation of multilayers can be delicate, complex and time-consuming. Peterson and collaborators achieved probe density control by varying the exposure time of the surface to the DNA solution, but also by modifying the buffer ionic strength and applying an attractive electrostatic field [9]. Although they claim to obtain highly reproducible data, their strategy involves direct immobilization of the probe on the surface, which could be unfavorable in several situations because of the formation of high background signals and/or loss of probe activity.

Here we propose a fast, simple and flexible method to regulate probe density immobilization starting from a single polymer precursor, an *N*, *N*-dimethylacrylamide-based copolymer, whose structure can be easily customized depending on the probe (DNA, proteins or peptides) that must be immobilized on the surface. These copolymers can form a stable and homogeneous functional nanometric coating on several materials (glass, silicon, gold, several thermoplastics, cellulose, nitrocellulose) [14,15,16,17] mediated by a combined physi-chemisorption mechanism [18,19]. 

Here we report a deep investigation into the role of functional group (either NAS or azide) concentration along the polymer chains on the assay performance, depending on the nature of the probe (protein or DNA) bound to the surface. To the best of our knowledge, all the studies reported in the literature only concern oligonucleotide probes, while the versatility of our approach allowed us to include proteins in this investigation. Polymers with increasing concentrations of NAS have been synthesized (and lately, modified with the azide group), then used in microarray assays with both protein (with different MWs) and oligonucleotide samples. We have also systematically examined and optimized the coating solution composition to obtain the most homogeneous surface for probe immobilization. 

This study allowed us to establish which formulation improves the test performance in specific applications. In particular, DNA microarrays are favored by higher concentrations of functional groups when the immobilization is driven by a click chemistry reaction. On the contrary, when using proteins, such as antibodies, surfaces with low functional group concentrations are to be preferred for better fluorescence performances. 

## 2. Materials and Methods

The reagents *N*, *N*-dimethylacrylamide (DMA), 3-(trimethoxylsilyl) propyl methacrylate (MAPS), 2,2′-Azodi(isobutyronitrile) (AIBN), rabbit IgG, α-lactalbumin, anhydrous tetrahydrofuran (THF), ammonium sulfate ((NH_4_)_2_SO_4_), phosphate buffered saline (PBS), and ethanolamine were purchased from Sigma Aldrich (St. Louis, MO, USA). The chemical 11-azido-3,6,9-trioxaundecan-1-azide was from Tokyo Chemical Industry Co., Ltd. (TCI; Zwijndrecht, Belgium).

*N*-acryloyloxysuccinimide (NAS) was synthesized as reported elsewhere [20].

Oligonucleotides for hybridization testing were synthesized by MWG-Biotech AG (Ebevsberg, Germany) and contained the following sequences: COCU8: 5′-NH_2_-GCCCACCTATAAGGTAAAAGTGA-3′, COCU10: 5′-Cy3-TCACTTTTACCTTATAGGTGGGC-3′. COCU10 was labeled with the fluorophore Cyanine 3. These oligonucleotides were freeze-dried and re-suspended in DI water at a final concentration of 100 μM before use. Untreated silicon slides 1000 Å Thermal Oxide (14 × 14 mm) were supplied by SVM, Silicon Valley Microelectronics Inc. (Santa Clara, CA USA) and were pretreated using a HARRICK Plasma Cleaner, PDC-002 (Ithaca, NY, USA) connected to an oxygen line. Contact angle measurements were collected via the sessile drop method using a CAM 200 instrument (KSV Ltd.), which utilizes video capture and subsequent image analysis. FT-IR spectra were registered using a Jasco-660 spectrometer and analyzed with Spectra Manager software 1.52 (Jasco, Easton, MD, USA).

### 2.1. Synthesis of MCP-NAS and MCP-N_3_ Copolymers 

**Figure 1:**
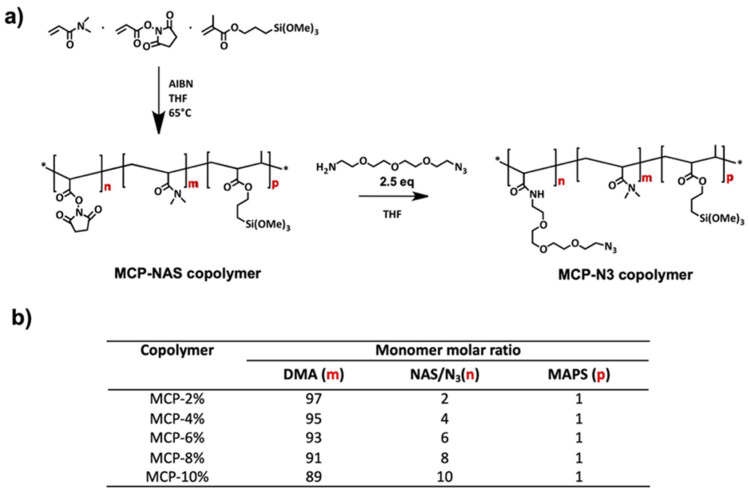
(**a**) Synthesis of MCP-NAS polymers and post-polymerization modification of NAS monomers with 2.5 equivalents of 11-Azido-3,6,9-trioxaundecan-1-amine to obtain the MCP-N_3_ derivatives; (**b**) composition of MCP copolymers expressed as monomer molar ratios; after the post-polymerization modification NAS groups are completely transformed into N_3_ groups; therefore, the final monomer molar ratio is identical.

Copolymer synthesis was performed as reported in [18,21]. Briefly, a 20% *w*/*v* total monomer concentration solution was prepared by dissolving *N*, *N*-dimethylacrylamide (DMA), *N*-acryloyloxysuccinimide (NAS) and 3-(trimethoxylsilyl) propyl methacrylate (MAPS) in anhydrous THF, following the molar ratios reported in Figure 1b. The solution was degassed for 20 min by purging argon into the monomer solution and then 2 mM of a thermoinitiator (AIBN) was added. The solution was heated for 2 h at 65 °C and kept under argon atmosphere. The solution was then diluted 1:1 with anhydrous THF and precipitated in petroleum ether. Polymers were collected as a white powder and dried under vacuum at room temperature.

For the post-polymerization modification of NAS with azide click groups, the protocol described in [18] was applied. Essentially the copolymer was dissolved in THF to a final concentration of 20% *w*/*v*; a 2.5 molar excess of 11-Azido-3,6,9-trioxaundecan-1-amine (with respect to the amount of NAS) was added to the crude material. The mixture was stirred for 5 h at room temperature and then diluted 1:1 with anhydrous THF. The polymers were precipitated in petroleum ether (using 10 times the volume of the reaction mixture), filtered using a Buchner funnel and dried under vacuum at room temperature.

The scheme of the synthesis and the copolymer composition is reported in Figure 1.

### 2.2. FT-IR and NMR Analysis

FT-IR spectra were collected on a Jasco 660 spectrophotometer with a computer running Spectra Manager 1.52 software. The samples (1 mg) were mixed with KBr (150 mg), after overnight drying under vacuum to remove water and humidity, and pressed to give a tablet. Then 32 scans were recorded over the range of 4000–400 cm^−1^ at a resolution of 4 cm^−1^ and intervals of 1 cm^−1^. The spectrum of a blank KBr tablet was subtracted using the Spectra Manager software.

MCP 4 % NAS, MCP 4% N_3_ and MCP4% NAS partially modified with the azide linker were characterized by acquiring ^13^C NMR spectra at 500MHz Bruker Avance II and 600MHz Bruker Avance DRX spectrometers equipped with a reverse 5mm TXI probe with a z gradient. About 30 mg of each polymer was dissolved in DMSO-d6 solvent. All spectra were recorded at 300K and calibrated on the solvent signal resonating 40.5 ppm (^13^C). Monodimensional ^13^C spectra were acquired with the inverse gated decoupling sequence to avoid the heteronuclear Overhauser effect, with sufficient relaxation delay to allow complete magnetization recovery among pulses. A spectral width of 37800 Hz was acquired over 64K points for carbon, while a width of 8012 Hz was acquired over 32K points for proton spectra.

### 2.3. Optimization of the Coating Solution Composition by Optical Density Measurements

Optical density measurements were performed in order to evaluate the correct amount of ammonium sulfate needed to prepare the polymer coating solution. The turbidity of the polymer solutions due to the addition of ammonium sulfate was measured using a MultiSkan SkyHigh microplate spectrophotometer (Thermo Scientific, Waltham, MA, USA) with the wavelength set at 600 nm. A 40 % saturation level, ammonium sulfate solution (1.8 M) was prepared by dissolving 242 g of ammonium sulfate in 1 L of DI water. This solution was then diluted to 1.2 M, 1 M, 0.8 M, 0.6 M, 0.4 M in solutions of the MCP-NAS and MCP-N_3_ polymers. As a control, solutions of pure polymer were analyzed as well. MCP 10% N_3_ was not considered further since it is completely insoluble in water.

### 2.4. Silicon Chip Coating

Silicon oxide slides were pretreated with oxygen plasma for 10 min: the oxygen pressure was set to 1.2 bar with a power of 29.6 W. Each copolymer was dissolved in DI water to a final concentration of 2% *w*/*v* and then diluted to a final concentration of 1% with different concentrations of ammonium sulfate solutions, depending on the polymer composition (see Table 1). The slides were immersed into each solution for 30 min, then rinsed with DI water, dried with a nitrogen stream and finally cured under vacuum at 80 °C for 15 min.

### 2.5. Functional Test of Coated Microarray Slides with Oligonucleotides

A 100 μM solution of the oligonucleotide 5′-GCCCACCTATAAGGTAAAAGTGA-3′, modified with a NH_2_ group (COCU8 NH_2_) or with a DBCO group (COCU8 DBCO) both in the 5′ position, was diluted to a final concentration of 50, 25, 10 and 1 μM in a 150 mM sodium phosphate buffer solution at pH 8.5 containing 0.01% *w*/*v* sucrose monolaurate. The oligonucleotides were deposited onto the surface using a non-contact microarray spotter (SCENION sci-FLEXARRAYER S12, Berlin, Germany) mounting an 80 μm nozzle. The spot volume, temperature and humidity were precisely controlled to 400 pL, 22 °C and 65%, respectively. Immediately after spotting, all the chips were stored overnight in a sealed chamber saturated with sodium chloride (40 g/100 mL H_2_O). After incubation, the silicon chips coated with the MCP-NAS polymers were treated with a pre-heated ethanolamine blocking solution (50 mM in 0.1 M TRIS/HCl buffer, pH 9 and kept at 50 °C for 15 min (this step is not required for MCP-N_3_ polymers). Consequently, all the chips were rinsed with DI water and immersed in a pre-heated solution containing 4X SSC (600 mM sodium chloride, 60 mM sodium citrate, pH 7.0) and 0.1% SDS and kept at 50 °C for 15 min before rinsing with DI water and drying with a nitrogen flow.

The printed chips were incubated with a complementary oligonucleotide target, 5′-Cy3-TCACTTTTACCTTATAGGTGGGC-3′ (COCU10) tagged with Cyanine 3 for fluorescence detection. COCU10 was diluted to a final concentration of 1 μM in an aqueous solution containing 2X SSC, 0.1% SDS and 0.2 mg/mL of BSA; 15 μL of this solution was layered onto the hybridization area and covered with a coverslip. The reaction was performed in a humid chamber at 65 °C for 2 h. Finally, the chips were washed in a 4X SSC solution at room temperature (to remove the coverslip) and then any unbounded oligonucleotide was removed using two successive rinses (5 min each) with a 2X SSC/0.1% SDS solution, pre-warmed at hybridization temperature (65 °C). A further two washes with 0.2X SSC and 0.1X SSC were carried out at room temperature for 1 min and, finally, the slides were dried using the nitrogen stream.

Fluorescent images of each chip were obtained using a confocal laser scanner (ScanArray Lite, Perkin Elmer) with the laser power set at 22% and the photomultiplier tube gain (PMT) at 64%, and analyzed using ScanArray Express software.

### 2.6. Functional Test of Coated Microarray Slides with Antibodies

Rabbit IgG dissolved in PBS at increasing concentrations (0.1, 1.5 and 10 mg/mL) was spotted on silicon chips coated with MCP-NAS polymers as reported in Section 2.5, using a non-contact microarray spotter (SCENION sci-FLEXARRAYER S12, Berlin, Germany) with an 80 μm nozzle. The spot volume, temperature and humidity were precisely controlled at 400 pL, 22 °C and 65%, respectively. Chips were stored in a humid chamber overnight at room temperature and blocked with ethanolamine blocking solution (see Section 2.5) for 1 h at room temperature. Chips were rinsed with DI water and dried with a nitrogen stream. All the slides were then incubated with a Cy3-labelled goat anti-Rabbit IgG 1 μg/mL in incubation buffer (50 mM Tris/HCl pH 7.6, 150 mM NaCl, 0.02% *w*/*v* Tween 20, 1 % *w*/*v* BSA) for 2 h at room temperature in a humid chamber. Slides were rinsed for 10 min in washing buffer (50 mM Tris/HCl pH 9, 250 mM NaCl, 0.05% *w*/*v* Tween 20) and then 10 min in PBS; finally, they were quickly rinsed with DI water and dried with a nitrogen stream. Fluorescent images of each chip were obtained using a confocal laser scanner (ScanArray Lite, Perkin Elmer) with the laser power set at 60% and the photomultiplier tube gain (PMT) at 60%, and analyzed using ScanArray Express software.

### 2.7. Functional Test of Coated Microarray Slides with Protein

PBS solutions with increasing concentrations of α-lactalbumin (0.1, 0.5, 1 mg/mL) were immobilized on silicon slides coated as reported in Section 2.5 and stored in a humid chamber at room temperature overnight. Chips were blocked with ethanolamine solution as previously reported and then incubated with goat anti α-lactalbumin IgG (1 μg/mL in incubation buffer, see Section 2.6) for 2 h at room temperature. Chips were rinsed with washing buffer (see Section 2.6) for 10 min and then with PBS for 10 min. The chips were then quickly rinsed with DI water and dried with a nitrogen stream. Chips were incubated with Cy3-labelled rabbit anti-goat IgG (1 μg/mL in incubation buffer, see Section 2.6) for 1 h at room temperature. Chips were rinsed with washing buffer for 10 min and then with PBS for 10 min. Then chips were quickly rinsed with DI water and dried with a nitrogen stream. All the slides were analyzed with a confocal laser scanner (ScanArray Lite, Perkin Elmer) with the laser power set at 60% and the photomultiplier tube gain (PMT) at 60%, and analyzed using ScanArray Express software.

## 3. Results and Discussion

The properties of a biosensor’s surface have a tremendous impact on its performance, since it greatly affects probe tethering and therefore the molecular recognition process between the two complementary molecules [22]. Surface coating mediated by polymers not only regulates passivation, eliminating non-specific binding, but also influences probe density, which has a great impact on the sensor’s sensitivity and specificity. However, the correspondence between probe density and assay performance is not always linear, since the nature of the probe plays its role itself causing steric and electrostatic effects which may alter the accessibility of the target. Moreover, the binding chemistry can have a role as well, influencing the conjugation yield and therefore surface saturation.

Our research group boasts much experience in the development of surface functionalization; this includes introducing a polymeric coating which works as a flexible scaffold for the customization of surface properties, such as hydrophilicity and charges [18,23]. In particular, surfaces functionalized with these polymers have been widely exploited for microarray applications using DNA [24,25,26], protein [27,28,29] and peptides [30,31], and have demonstrated superior characteristics compared with other existing methods [29,32]. In addition, their combination with a proper substrate material—in particular, silicon with a 100 nm thermally grown oxide layer—enhances the fluorescence signal, thanks to a constructive optical interference phenomenon [15]. 

The basic structure (reported in Figure 1) is composed of *N*, *N*-dimethylacrylamide (DMA), which confers an amphiphilic characteristic to the polymer and forms weak non-covalent interactions (hydrogen bonds, Van der Waals, hydrophobic forces) with the surface; these are reinforced by the presence of 3-(trimethoxysilyl) propyl methacrylate (MAPS), which establishes covalent bonds with surface silanols. The third ingredient, *N*-acryloyloxysuccinimmide (NAS), is a chemically reactive monomer that covalently binds amine by a nucleophilic substitution (SN2) reaction, therefore promoting the immobilization of proteins, peptides and amine-modified oligonucleotides. Furthermore, it enables the insertion of other functional groups which supports alternative conjugation chemistries, such as the so called *click chemistry* reactions. In particular, the azido groups introduced by post-polymerization modification allow the so-called Strain-promoted alkyne–azide cycloaddition (SPAAC) reaction with dibenzocyclooctyne (DBCO) bearing molecules. This particular click chemistry reaction, by definition, does not require any particular condition (pH, solvent, temperature) to be executed, and in particular, does not necessitate any catalyst. The absence of a catalyst is a great advantage because, very often, catalysts, such as copper, can be cytotoxic and therefore not suitable for biological systems. Moreover, the DBCO-promoted reaction is faster and spontaneous compared to the classic copper-catalyzed azide–alkyne cycloaddition [33].

The coating, obtained by simply immersing the support into a diluted aqueous solution of the polymer, provides a homogeneous and uniform nanometric functional film over the entire support as demonstrated by AFM analysis and by an interferometric fluorescence spectroscopy technique called Spectral Self-Interference Fluorescence Microscopy (SSFM) [32,34,35]. Water contact angle measurements, performed in different areas of the coated chip, also support the homogeneity of the coating [18].

Over the years we have exploited the flexibility of this polymer scaffold with the aim of adapting and tailoring surface properties, but we have never optimized the coating protocol nor explored the ability to tune the probe density by taking into account the nature of the probe itself. Therefore, we here report a systematic investigation into the role of the concentration of the functional groups to establish the correct amount needed to enhance the performance of specific microarray applications. Considering the solubility modification generated by the increase of functional monomer concentration, we have firstly optimized the coating procedure by analyzing the appropriate amount of the additive (in particular, ammonium sulfate) to be added to the coating solution to obtain a homogeneous film on the microarray support. Subsequently, we have analyzed the performance of the coated surfaces by immobilizing probes of different natures (DNA oligonucleotide and proteins) at different concentrations to establish the perfect polymer formulation and conditions to be applied, depending on the sensing application.

Finally, we have also examined if the modification of NAS into click functional groups could lead to potential differences, both in terms of coating protocol and probe grafting.

### 3.1. FT-IR Analysis

**Figure 2:**
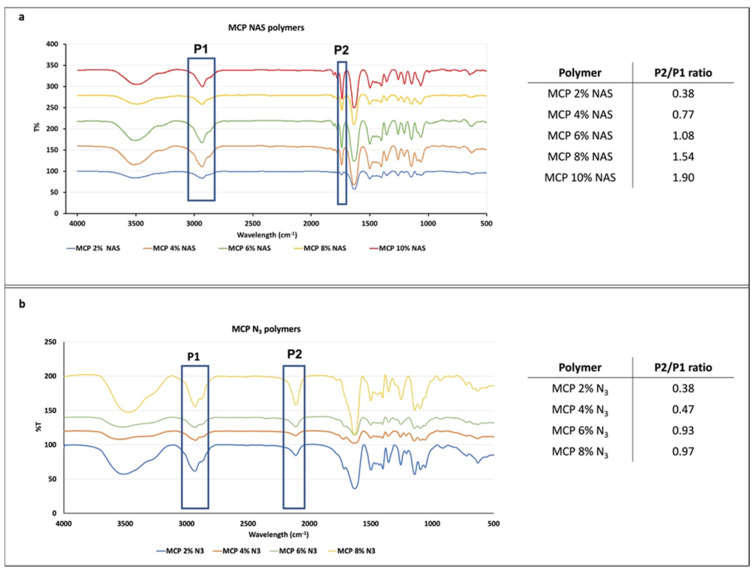
FT-IR spectra of MCP polymers with increasing concentrations of (**a**) NAS or (**b**) azide monomer. The blue rectangles in (**a**) highlight the peak at 3000 cm^−1^ and 1740 cm^−1^ (corresponding to DMA carbonyl stretching and the NAS ester bond stretching, respectively), while those in (**b**) highlight the 3000 cm^−1^ and 2100 cm^−1^ peaks (corresponding to DMA carbonyl stretching and N_3_ stretching, respectively). The ratio between the height of these two peaks (P2/P1) gives indications regarding the concentration of the functional monomer along the polymer chain.

FT-IR analysis was performed to evaluate the presence of NAS or azide monomers and their increase in the different formulations. To this end, we evaluated the ratio between the height of two peaks, one corresponding to the functionality introduced at increasing concentrations and one corresponding to the polymer backbone. Since the latter does not vary, the increase in the ratio of the two peaks’ heights is due to the increase in the molar fraction of the NAS or azide group introduced along the polymer chain. NAS exhibits a specific signal at 1740 cm^−1^ corresponding to the stretching of the ester bond, while azide shows a peak at 2100 cm^1^. Different batches of MCP polymers, containing increasing concentration of NAS or azide monomer, have been analyzed and their spectra are shown in Figure 2. As can be observed, all the spectra show the expected specific signals. While FT-IR analysis is not quantitative, the intensity of the ester and azide bond signal (highlighted in the blue rectangle, P2, Figure 2a,b) increases with the addition of NAS or azide into the polymer chain. The ratio between the height of this peak (P2) and the height of the peak at 3000 cm^−1^ (P1), corresponding to stretching of DMA carbonyl backbone, was measured and values are reported in the tables in Figure 2a,b. As expected, the ratio increases linearly, demonstrating that the incorporation of NAS along the polymer chain is higher when its concentration is higher in the reaction feed (for example, MCP 10% NAS versus MCP 2% NAS). The same trend can be observed for the MCP-N_3_ polymers, suggesting the proper introduction of the azide moiety by post-polymerization modification and the quantitative yield of this reaction. Differences in the ratio values between MCP-NAS and MCP-N_3_ polymers must be ascribed to the distinct stretching intensity of the ester and azide bonds. 

**Figure 3:**
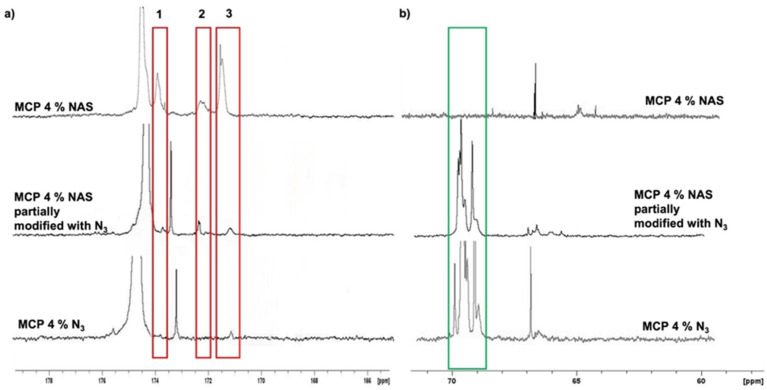
^13^C spectrum of the (**a**) carbonyl and (**b**) aliphatic region of MCP 4%NAS, MCP 4%NAS partially modified with azide groups, and MCP 4% N_3_. (**a**) The signals in the red rectangles correspond to the carbonyls of the succinimide group (NAS). It can be observed that these peaks disappear in the spectrum of MCP 4% N_3_ since the NAS groups are totally substituted by azide moieties, demonstrating the absence of impurities, which remain in the case where the conversion is not completed (MCP 4%NAS partially modified). (**b**) The peak in the green rectangle represents the PEG signal which is absent in the MCP 4% NAS, while it appears when the 11-azido-3,6,9-trioxaundecan-1-amine linker is inserted in the polymer chain to convert NAS into azide groups.

The evaluation of remaining impurities, due to an incomplete conversion of NAS into azide groups, was evaluated by NMR. We compared the ^13^C spectra of MCP 4% N_3_ with that of its parent polymer, MCP 4% NAS (Figure 3). The red rectangles highlight the most representative signals (ranging from 176 to 168 ppm) belonging to the carbonyl region. In this region (Figure 3a), the three signals belonging to the NAS carbonyls of MCP 4% NAS—occurring at 174 ppm (peak 1), 172 ppm (peak 2) and 171 ppm (peak 3)—totally disappeared in the MCP 4% N_3_ spectrum (Figure 3a). The complete conversion of NAS into azide groups is demonstrated by comparing the spectrum of MCP 4% azide with the spectrum of a MCP 4% NAS partially modified with azide groups: in this case, it can be observed that the peaks corresponding to the NAS carbonyls are still present, while they totally disappear if the conversion into azide groups is complete. The partial modification of the copolymer was obtained by reducing the quantity of the azide linker during the post-polymerization modification reaction, specifically, by using 1.2 equivalents of the 11-azido-3,6,9-trioxaundecan-1-amine instead of 2.5 equivalents which assures a complete conversion of the NAS groups. 

Figure 3b shows the aliphatic region. The peak highlighted in the green rectangle in this area represents the signals of the PEG chain of the 11-azido-3,6,9-trioxaundecan-1-amine linker which is used during the post-polymerization modification to convert the NAS into azide groups. As can be observed, this peak is totally absent in the spectrum of the MCP 4% NAS polymer, while it is present in the spectra of the azide-modified polymers. 

### 3.2. Optimization of the Coating Solution Composition by Optical Density Measurements

The amphiphilic nature of MCP polymers, conferred by the DMA backbone, assures high solubility in water; therefore, the coating of surfaces with MCP polymers requires the addition of ammonium sulfate. This salt is fundamental to obtain a proper and homogeneous coating on the chip surface, because it slightly reduces the solubility of the polymer in water, favoring adsorption on the support (*salting out* effect). The absence of this additive results in weak, non-homogeneous and non-reproducible coating or, in some cases, it prevents the formation of the coating itself (data not shown). However, the concentration of this salt must be finely regulated; if used in excess, it causes massive precipitation of the polymer, easily visible as a drastic change in the solution transparency. Polymer precipitation in the coating solution would lead to uneven coating and formation of stains on the surfaces, negatively affecting the immobilization of probes and generating background signal.

**Figure 4:**
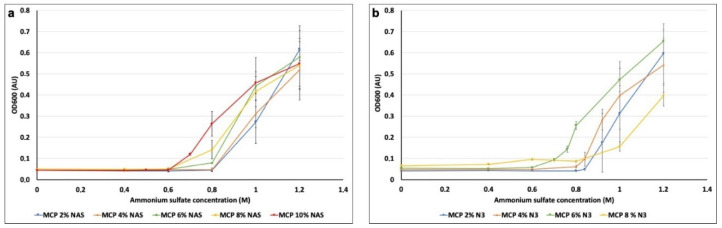
Variation of OD_600_ measured for each MCP polymer bearing (**a**) NAS functionality and (**b**) azide groups after the addition of increasing amounts of ammonium sulfate.

The increment of NAS and azide functions to the polymer composition slightly modifies overall polymer solubility when ammonium sulfate is added; therefore, it is necessary to establish the correct concentration of salt to be added to each coating solution to avoid the issues described above. Although at certain concentrations the induced turbidity is clearly visible to the naked eye, at intermediate concentrations it is not discernible. Consequently, we have exploited optical density to measure the degree of turbidity induced by the incremental addition of ammonium sulfate to the polymer solution. Optical density (OD_600_), is a property that describes a material’s ability to absorb the power of a given light that is passed through that material. It is a UV technique commonly used to measure microbial growth since particles in solution scatter light, and the more particles can be found in a solution, the more light is scattered by them [36]. To this extent, we prepared several solutions of each MCP polymer (at a fixed 1% *w*/*v* concentration), added increasing concentrations of ammonium sulfate (0, 0.4, 0.6, 0.8, 1, 1.2, 1.4 and 1.6 M) and measured the turbidity with a spectrophotometer with the wavelength set at 600 nm. We performed this experiment for all the polymer formulations so as to evaluate the maximum amount of ammonium sulfate which does not cause any polymer precipitation. Results are depicted in Figure 4.

Variations in OD due to the addition of ammonium sulfate occurred in the range between 0.6 and 0.8 M of ammonium sulfate for all polymer solutions. In particular, MCP 2% NAS and MCP 4% NAS showed a significant increase of OD values when 1 M of ammonium sulfate was added to the polymer solutions, while for MCP 6% NAS, MCP 8% NAS and MCP 10% NAS, the variation occurred when 0.6 M of ammonium sulfate was added. Similar results were obtained with the azide bearing copolymers, demonstrating that the modification of the functional monomer (azide instead of NAS) does not affect overall polymer solubility. Concentrations of ammonium sulfate higher than 1 M did not report reliable data because of the saturation of the spectrophotometer detector (at 1.6 M, the signal drops due to massive polymer precipitation, data not shown). 

Observing the range of concentrations between 0.6 and 0.8M (Figure 4a,b), slight differences among all polymers can be detected. We therefore analyzed intermediate concentrations of ammonium sulfate to determine the exact quantity to be used to avoid polymer precipitation. Following this procedure, we have established the correct amount of ammonium sulfate to be added to the coating solution of each polymer, reported in Table 1.

### 3.3. Functional Test of Coated Microarray Slides with Oligonucleotides

**Figure 5:**
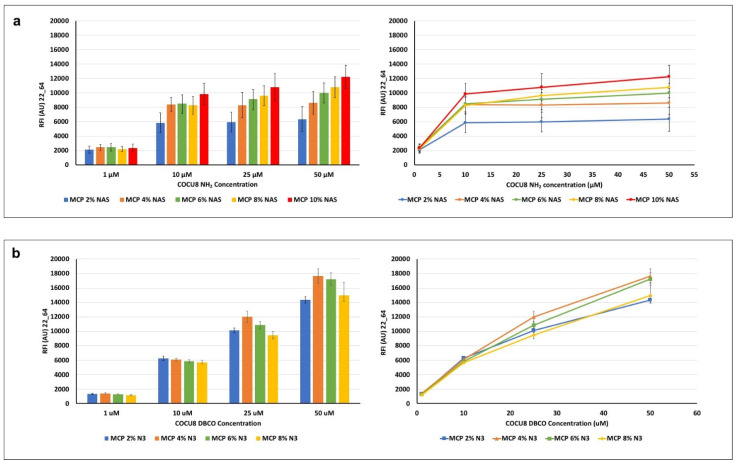
Fluorescence intensity signals obtained after hybridization of an immobilized oligonucleotide with its Cy-3 labelled complementary strand. The (**a**) amino-modified or (**b**) DBCO-modified oligonucleotide probe was immobilized at increasing concentrations (1, 10, 25, 50 μM) on surfaces coated with MCP polymers bearing increasing concentrations of (**a**) NAS, a monomer able to covalently bind amine probes; or (**b**) azide groups which react with triple bonds. Fluorescence was measured with a confocal laser scanner with power set at 22% and the photomultiplier tube gain (PMT) at 64%. The reported signal intensities are the average of the Relative Fluorescence Intensity (RFI) of each 10 × 2 subarray, each corresponding to a single oligonucleotide concentration. Therefore, the error bars are the standard deviations of the fluorescence intensity of 20 spots.

Silicon slides coated with MCP polymers with increasing concentrations of NAS and azide monomer (using the correct amount of ammonium sulfate, depending on the concentration of NAS and azide; see Table 1) were used to test differences in DNA microarray assay performance due to the different polymer formulations. For this purpose, a 23-mer oligonucleotide was immobilized on the coated surfaces at increasing concentrations (1, 10, 25, 50 μM) and then hybridized with 1 μM of the fluorescently labelled complementary strand. Results of the average fluorescent intensity signals, measured with a confocal laser scanner for each coated chip, are reported in Figure 5.

In general, it can be observed that, at equal concentrations of the spotted probe, higher concentrations of the NAS monomer generate a higher fluorescence signal (MCP 10% NAS vs. MCP 2 % NAS, Figure 4a), suggesting that the ability to immobilize a higher density of probes on the surface, available for target interactions. This is particularly relevant when the concentration of the probe on the surface is high (50 μM vs. 1 μM). Intermediate concentrations of NAS monomer do not show significant differences, although there is an increasing trend, particularly remarkable for higher probe concentrations. 

A similar behavior is observed among the MCP-N_3_ polymer family, thus demonstrating the effectiveness of the MCP-NAS polymers to serve as a scaffold for the generation of alternative surface chemistries. However, the azide surfaces show an increasing trend of fluorescence signal when higher probe concentrations are immobilized on the surface (1 μM vs. 50 μM): this does not happen on MCP-NAS coated slides, which, on the contrary, tend to reach a signal plateau. This different behavior can be due to the yield and velocity of the click reaction versus the nucleophilic substitution reaction which happens between NAS and amine. This last reaction is in fact slower than the click reaction between azide and triple bonds and it is negatively affected by hydrolysis, which drastically reduces the binding sites for the amine probes on the surface. As a consequence, saturation of these binding sites is obtained at lower probe concentration. Click reactions have a very fast kinetic, and do not suffer from hydrolysis, hence more probes can be conjugated to the surface, resulting in increasing fluorescence intensity signals. A difference in bound mass between the two chemistries has been demonstrated in a recent work where we immobilized amine or DBCO-modified oligonucleotides on surfaces coated with MCP 10% NAS and MCP 10% N_3_ [37]. Label-free images of the dry-blocked silicon chips using an interferometric technique called Interferometric Reflectance Imaging Sensor (IRIS) highlighted that when increasing the spotting concentration on MCP 10% NAS-coated chips, the available binding sites are saturated for concentrations higher than 10 μM (the mass immobilized at 25 μM is fairly similar to the one immobilized at 50 μM). Conversely, on a MCP 10% N_3_ coated surface, a linear increase in mass density was observed with the concentration increase. However, these data are not completely in line with the fluorescence results shown in Figure 5, particularly with regard to the MCP-N_3_ copolymers’ behavior. In fact, this particular series of MCP-N_3_ copolymers have been obtained using an azido linker comprised of a short polyethylene glycol (PEG) chain, while the copolymer analyzed in our previous work [37] was synthesized using a shorter linker, 3-azido-1-propylamine [18]. The choice of changing the azide linker during the post-polymerization modification was due to safety reasons, since it is widely known that short chain azide reagents may cause explosions when used in high concentrations. The increase of azide groups unavoidably causes an increase in the PEG chains, which have an effect on the adsorption of the copolymer on the support, and therefore on its conformation when on the surface. These conformational changes very likely negatively affect and prevents the interaction of the target with the tethered probe. This might be the reason why, the concentration of immobilized oligonucleotides being equal, the signal of MCO 4% N_3_ is higher that the signal of MCP 8% N_3_. On the contrary, the increase of bound probe is demonstrated by the linear increase of fluorescence signals obtained for all MCP-N_3_ polymers by immobilizing higher concentration of oligonucleotide (10 μM vs. 50 μM).

Overall, these results suggest that MCP-N_3_ polymers are more efficient in terms of immobilization, since higher fluorescence signals can be reached by immobilizing higher probe concentrations, consequently, MCP-N_3_ polymers (in particular, MCP 4% N_3_) must be preferred when high concentrations of DBCO probes (25 or 50 μM) are available. Conversely, among the MCP-NAS copolymers, the one bearing 10% of NAS functionality (MCP10% NAS) offers the higher fluorescence signals; however, it should be taken into account that saturation of the binding sites is reached using a concentration of amino-modified probe of 10 μM.

### 3.4. Functional Test of Coated Microarray Slides with Antibodies and Small Mw Proteins

The behavior of the different coated surfaces was tested with different probes, in particular, immunoglobulins and small proteins. In this case, only MCP-NAS polymers were tested since DBCO-modified antibodies do not exist in nature and require specific and time-consuming modification reactions. IgG antibodies are large molecules, having a molecular weight of approximately 150 kDa, with several functionalities which can easily react with the surface and bind through electrostatic or hydrophobic interaction, hence their correct and oriented conjugation to the surface has a great impact on the overall assay performance. In fact, random immobilization and lack of orientation may cause the impossibility of interaction with the target and, in the worst cases, loss of activity due to structure denaturation. Moreover, because of their dimensions, high density of probe immobilization may be detrimental because of steric hindrance issues. 

**Figure 6:**
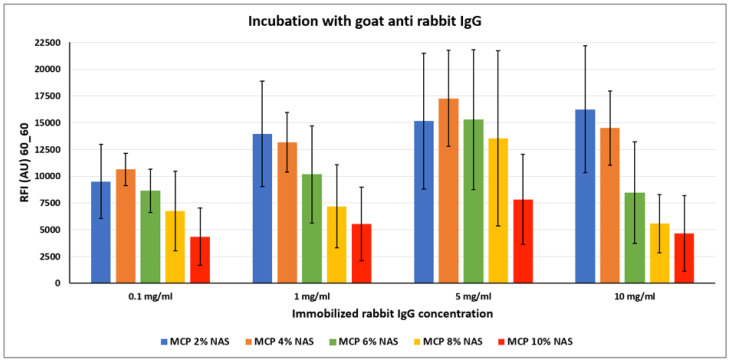
Fluorescence intensity signals obtained on silicon slides coated with MCP NAS polymers, spotted with increasing concentrations of rabbit IgG and incubated with a Cy3-labelled goat anti-rabbit IgG. Fluorescence was measured with a confocal laser scanner with power set at 60% and the photomultiplier tube gain (PMT) at 60%. The reported signal intensities are the average of the Relative Fluorescence Intensity (RFI) of each 10 × 2 subarray, each corresponding to a single rabbit IgG concentration. Therefore, the error bars are the standard deviations of the fluorescence intensity of 20 spots.

In order to investigate the most suitable surface coating for the immobilization of antibodies, we spotted increasing concentrations of a rabbit IgG on silicon slides coated with the MCP-NAS polymer family. Each slide was then incubated with a secondary antibody against rabbit IgG (goat anti-rabbit) that had been labelled with Cy3 for fluorescence detection. Results are shown in Figure 6.

Contrary to what is observed with DNA probes, immobilization of antibodies benefits from lower functionality concentration on the surface. In fact, higher fluorescence signals were obtained on MCP 2 % NAS and MCP 4% NAS, suggesting that a higher concentration of functional groups might cause density issues, as described above. One possible explanation could be that antibodies are not properly retained on the surface because of crowding effects and are released unevenly during the washing and rinsing steps. This phenomenon is emphasized when high concentrations of probes are immobilized on the surface as demonstrated by the error bars values obtained when spotting 5 and 10 mg/mL of rabbit IgG, deleteriously affecting the performance of the assay. 

**Figure 7:**
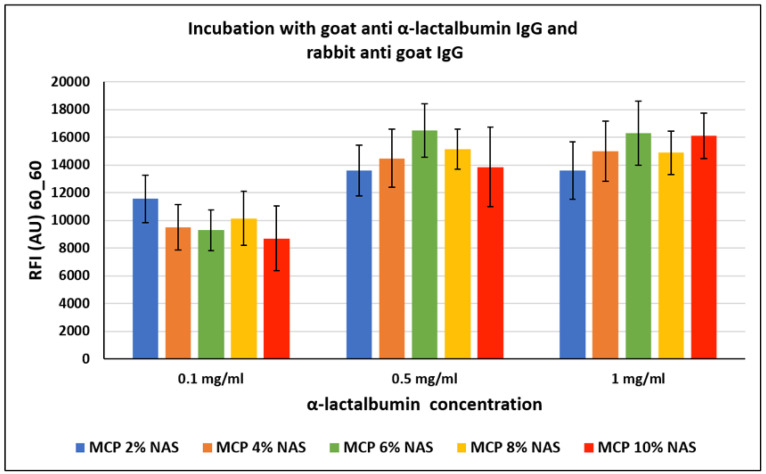
Fluorescence intensity signals obtained on silicon slides coated with MCP NAS polymers, spotted with increasing concentrations of α-lactalbumin and incubated with a goat α-lactalbumin IgG and then with a Cy3-labelled rabbit anti-goat IgG for fluorescence detection. Fluorescence was measured with a confocal laser scanner with power set at 60% and the photomultiplier tube gain (PMT) at 60%. The reported signal intensities are the average of the Relative Fluorescence Intensity (RFI) of each 10 × 2 subarray, each corresponding to a single protein concentration. Therefore, the error bars are the standard deviations of the fluorescence intensity of 20 spots.

The crowding effect theory is supported by the fact that, surprisingly, this event does not occur with all proteins, especially in the case of small proteins. In particular, α-lactalbumin, a small protein mainly found in milk with a molecular weight of about 15 KDa, was similarly immobilized on silicon slides coated with MCP polymers (as reported in Section 2.4). The chips were then incubated with a goat α-lactalbumin IgG and then with a Cy3-labelled rabbit anti-goat IgG for fluorescence detection. Fluorescence intensity results are reported in Figure 7. In this case, the increasing concentration of NAS functionality does not seem to have a significant effect on the assay performance, demonstrating that the NAS concentration does not affect the probe immobilization modality.

In both cases, lack of dependence between the increase in polymer functionalities and fluorescence signal could also be due to an increment of protein interactions with the surface, causing diminution of the freedom of protein movement and inducing a stiffening of the protein structure, thereby hindering the interaction with its counterpart in solution. Although it is widely known that immobilization of proteins can also occur by different mechanisms which could affect the protein’s activity (such as electrostatic or hydrophobic interactions), this is not the case. In particular, we tried to immobilize a rabbit IgG on a surface coated with a polymer bearing acrylic acid instead of NAS moieties (acrylic acid is the remaining functionality after complete NAS hydrolysis) and we observed negligible bio-probe conjugation (data not shown).

## 4. Conclusions

We have explored different formulations of DMA-based polymers with the aim of correlating the concentration of functional groups with the performance of a microarray assay, depending on the type of probe which must be immobilized.

To this end, we synthesized polymers with increasing concentrations of NAS monomer and, by post-polymerization modification, the NAS functionality was transformed into azide moieties which perform click reaction-based conjugation with triple bonds-modified probes. Incorporation of increasing amounts of NAS monomer was checked using FT-IR, which also confirmed the quantitative conversion into azide functionality by post-polymerization modification of all the formulations.

The addition of functional monomer slightly modified the polymer solubility; therefore, the concentration of ammonium sulfate, used to obtain a homogeneous coating, was optimized for each formulation. Indeed, the choice of the correct amount of salt to be added to the polymer coating solution assures homogeneous coating with reduced background.

The polymers have been tested by coating silicon surfaces to perform microarray assays using oligonucleotide probes and proteins with different MWs. In general, DNA microarrays are favored by higher concentrations of functional groups because this results in higher probe density, which allows easy recognition and binding to the target in solution. On the contrary, high MW proteins, such as antibodies, suffer crowding effects; therefore surfaces with a low concentration of functional groups are to be preferred for better fluorescence performances. 

## Figures and Tables

**Table 1:** Molar concentration of ammonium sulfate used to obtain optimal polymer coating solution depending on the concentration of NAS and azide monomer.

Polymer	Ammonium Sulfate Concentration (M)
MCP 2% NAS; MCP 2% N_3_; MCP 4% N_3_	0.8
MCP 4% NAS	0.7
MCP 6% NAS; MCP 6% N_3_	0.6
MCP 8% NAS	0.5
MCP 10 % NAS; MCP 8% N_3_	0.4

## Data Availability

Not applicable.

## Abbreviations

DMA	N, N-dimethylacrylamide
NAS	N-acryloyloxysuccinimide
MAPS	3-(trimethoxylsilyl) propyl methacrylate
OEG	oligo(ethylene glycol)
N3	azide; PBS
PBS	phosphate buffered saline
FT-IR	Fourier-transform infrared spectroscopy
OD	optical density
PLL	poly-(l-lysine)
PSS	poly(styrene sulfonate)
PEG	Polyethylene glycol
